# A novel homozygous variant in *SLC25A46* gene associated with pontocerebellar hypoplasia type 1E: a case report

**DOI:** 10.3389/fped.2024.1303772

**Published:** 2024-02-23

**Authors:** Adrien Guillaume, Vojtech Stejskal, Guillaume Smits, Dorottya Kelen

**Affiliations:** ^1^ Neonatal Intensive Care Unit, Hôpital Universitaire de Bruxelles, Université Libre de Bruxelles (ULB), Brussels, Belgium; ^2^ Center of Human Genetics, Hôpital Universitaire de Bruxelles, Université Libre de Bruxelles (ULB), Brussels, Belgium

**Keywords:** neonatal encephalopathy, pontocerebellar hypoplasia type 1E, *SLC25A46*, clinical diagnosis, therapeutic strategies

## Abstract

Neonatal encephalopathy (NE) is a complex clinical condition with diverse etiologies. Hypoxic-ischemic encephalopathy (HIE) is a major contributor to NE cases. However, distinguishing NE subtypes, such as pontocerebellar hypoplasia type 1E (PCH1E), from HIE can be challenging due to overlapping clinical features. Here, we present a case of PCH1E in a neonate with a homozygous mutation c.72delT p. (Phe24LeufsTer20) in the *SLC25A46* gene. The severity of PCH1E associated NE highlighted the significance of early recognition to guide appropriate clinical management.

## Introduction

Neonatal encephalopathy (NE) is characterized by disruption of brain function, manifested through altered consciousness, accompanied by seizures, cardiorespiratory compromise, or abnormal tone and reflexes (1).

Hypoxic-ischemic encephalopathy (HIE) accounts for half of the cases of NE and typically arises from acute hypoxic-ischemic insults during the intrapartum perinatal period. The other etiologies of NE include acquired conditions (e.g., congenital infections, anemia, stroke), genetic factors, neurometabolic disorders, and cardiac anomalies (2).

Initial management of suspected HIE imply resuscitation, intensive care, and diagnostic assessments to determine the extent of brain injury. When HIE diagnosis is uncertain or excluded, second line investigations are needed to identify alternative etiologies and tailor appropriate therapeutic strategies.

This report presents a case of pontocerebellar hypoplasia (PCH), a rare disorder linked to NE. PCH is a group of autosomal recessive disorders characterized by neurodegeneration of the cerebellum and brainstem, leading to severe neurodevelopmental delays and early mortality. Once categorized into two clinical subtypes (3), advancements have refined the classification to differentiate 16 subtypes, each correlated with specific genetic anomalies (4). The incidence of each subtype remains undetermined except for the most frequent, PCH2A, which is estimated to be lower than 1/200.000 (5).

Distinct from other PCH forms, PCH type 1 exhibits additional central and peripheral motor dysfunction along with anterior horn cell degeneration similar to spinal muscular atrophy (6). Although cerebellar hypoplasia is a consistent feature in PCH type 1, the involvement of the ventral pons and cerebrum is variable (7).

Presently, PCH type 1 comprises six subcategories (PCH1 A-F), with PCH1E (OMIM 610826) linked to mutations in the *SLC25A46* gene, representing the most severe form (8). SLC25A46, a mitochondrial carrier protein belonging to the SLC25 family, has been implicated in neurodegeneration. Animal models have demonstrated the protein's engagement with inner mitochondrial membrane remodeling, with disruptions in mitochondrial dynamics in mutated zebrafish neurons (9, 10). Biallelic mutations in the *SLC25A46* gene are also associated with hereditary motor and sensory neuropathy type 6B (HMSN6B), a less severe condition displaying overlapping features with PCH1E (10). Wan et al. determined that mutations leading to a significant reduction in SLC25A46 levels are responsible for PCH1E, the most severe clinical presentation among the spectrum of SLC25A46-related diseases (8).

This report highlights the distinct clinical features of PCH1E, characterized by early-onset severe hypotonia and respiratory insufficiency leading to death within weeks of birth (11). Currently, diagnostic methods for early PCH1E detection remain unavailable. Thus, it is crucial for neonatologists to consider genetic or metabolic causes of neonatal encephalopathy, fostering a proactive approach in investigating potential underlying conditions. While encountering patients with PCH1E may be rare, a broader understanding of various etiologies beyond hypoxia is essential for informed clinical decision-making and accurate diagnosis in cases of neonatal encephalopathy.

## Case presentation

We report the case of a term male infant born to second-degree consanguineous Syrian parents, with a history of one sibling's neonatal death of unknown etiology. No antenatal concerns such as reduced fetal movements, contractures or polyhydramnios have been reported, an elective Caesarean section for breech presentation was performed at 38 weeks of gestation in a local hospital.

At birth, the newborn unexpectedly presented with profound hypotonia, bradycardia (HR <60 beats/minute), and absent spontaneous respiratory efforts, with APGAR scores of 1, 3, and 5 at 1, 5, and 10 min respectively. Umbilical arterial blood gas analysis showed mixed acidosis (pH 7.10, pCO2 64 mmHg, pO2 59 mmHg, lactate 5.5 mmol/L, base excess −11 mmol/L). Physical examination revealed no dysmorphic features, a birth weight of 3,610 g (P50-90), length of 52 cm (P90), and head circumference of 34 cm (P 90) according to Fenton's growth charts (12).

Prompt resuscitation was performed according to the newborn life support guidelines (13), involving positive pressure ventilation, chest compressions, and endotracheal intubation. Physical examination showed lethargy, severe hypotonia, absent Moro's and sucking reflexes, and weak grasping ability. Irregular respiratory patterns with apnea and hyperventilation were observed. Given of the discordance between the neurological status of the patient and the lack of severe metabolic acidosis combined with the absence of sentinel event cooling was not started. The neonate then was referred and transferred to our level 3 neonatal intensive care unit (NICU) with stable vital parameters. Clinical improvement was observed upon arrival, efficient respiratory efforts prompted transition to high-flow nasal cannula at 6 L/min. Laboratory investigations revealed no evidence of multiorgan involvement. Capillary lactate levels normalized from birth to 6 h of life. In view of improving conditions the decision of normothermia was maintained. However, mechanical ventilation was restarted at eight hours of life due to recurrent apneas and worsening hypotonia.

First line assessments included cranial ultrasound indicating bilateral mild periventricular hyperechogenicity, and continuous video- electroencephalography (EEG) of 24 h. The trace was moderately discontinuous, mainly synchronous, labile and reactive. An abnormal low voltage background likely suggested global cortical dysfunction. Several dysmature patterns were noted: no cycle distinction between wakefulness and sleep, persistent delta brushes, and discontinuous pattern all along the trace, with 5–10 s of interburst interval as expected at 34 weeks. Bursts were composed by multirythmic activities and frequent frontal rhythmic delta activities. No epileptiform discharges. No seizure was recorded. These features are indicative of mild to moderate encephalopathy. Absence of seizures was noted. Antibiotics were stopped following negative blood culture and C-reactive protein results after 48 h.

**Figure 1:**
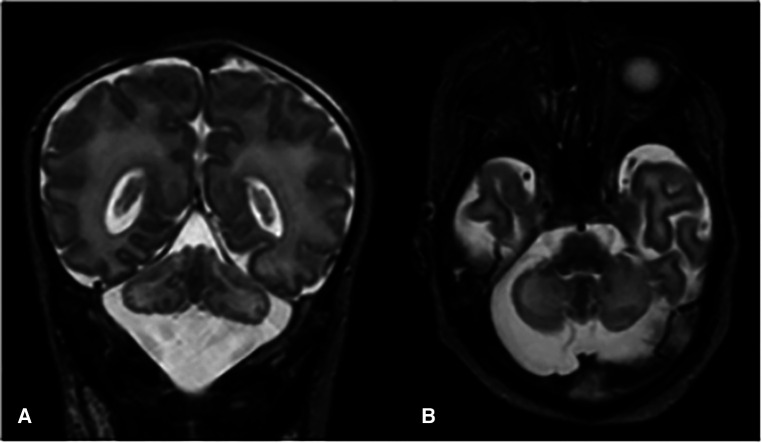
Coronal (**A**) and axial (**B**) T2-weighted images show global cerebellar hypoplasia with symmetric involvement of both hemispheres and vermis, along with a very large cisterna magna. There was no obvious abnormality of the brainstem. The cerebellar folia and fourth ventricle were not dilated, indicating there was no volume loss. The cerebellar foliation pattern was normal and there were no heterotopic nodules of gray matter, indicating there was no dysplasia.

**Figure 2:**
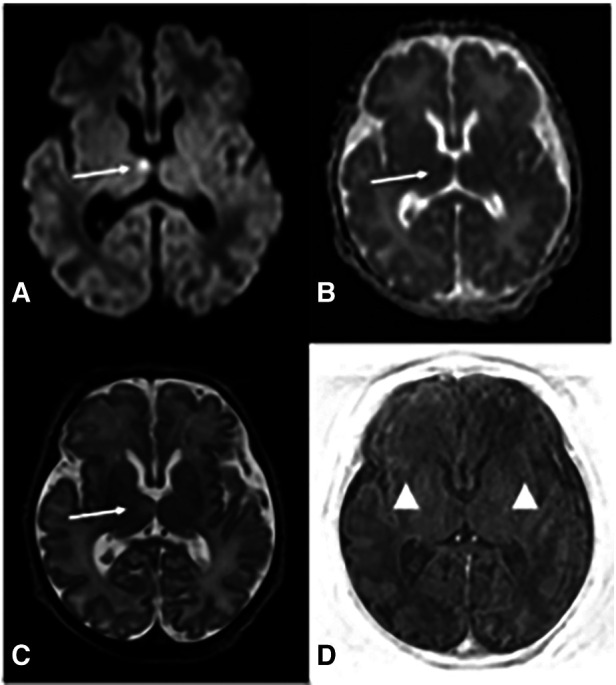
Brain MRI at d6 demonstrates deep gray and white matter hypoxia. Axial diffusion-weighted (DWI) image (**A**) reveals increased b1000 signal in the right ventro-medial thalamus and corresponding decreased apparent diffusion coefficient (ADC) (**B**) (*arrows in* **A** and **B**) indicating restricted diffusion. There is associated pathological T2 prolongation (*arrow in* **C**). Axial TI|R-weighted image (**D**) shows lack of the physiological T1 hyperintensity of posterior limb of internal capsules (PLIC) (*arrowheads*), whereas PLIC is usually hyperintense on T1 with respect to the thalami and basal ganglia.

Absence of neurological improvement prompted extended metabolic and endocrine evaluations: the levels of serum amino acids, sialotransferrin, TSH, free T4, cortisol, ammonia, and 17 OH progesterone were found to be within normal range. Urine amino acid analysis detected mild elevation (11 mmol/mol creatinine) in excretion of 3-methylglutaconic acid, implying possible mitochondrial dysfunction (14). Brain MRI on day 6 showed cerebellar hypoplasia with symmetric involvement of the vermis and hemispheres, without dysplasia (Figure 1) and a potential ischemic thalamic lesion (Figure 2).

At two weeks of life, electrophysiological studies (sensory and motor nerve conduction and electromyography) revealed severe sensorimotor neuropathy. The newborn was managed with supportive care, and died to respiratory insufficiency on day 18. Post-mortem examination was declined in accordance with parental wishes.

Subsequent exome sequencing unveiled a homozygous mutation c.72delT p. (Phe24LeufsTer20) in the *SLC25A46* gene, with both parents identified as heterozygous carriers.

## Discussion

PCH1E is marked by a grave clinical course, with most patients dying within the first month of life at neonatal intensive care units (3, 9–11, 15–20). Prompt and accurate diagnosis remains challenging, as the definitive confirmation through exome sequencing often arrives after life-sustaining decisions have been made.

Recognizing neuromuscular diseases such as PCH1E within the broad spectrum of NE is imperative for facilitating management and appropriate palliative care decisions. Impaired postnatal adaptation, as evident in inefficient respiration and severe hypotonia, compares with typical neonatal encephalopathy caused by perinatal asphyxia (10, 11, 17, 18). An important decision emerged in the management of the presented case, as the medical team opted not to initiate cooling therapy in the face of neonatal encephalopathy of unknown origin. This decision, while supported by certain clinical indicators such as the discrepancy between the severity of clinical presentation at birth and absence of fetal distress or sentinel events, coupled with the lack of severe acidosis at birth, remains subject to controversy. Conventionally, when faced with neonatal encephalopathy and uncertainty regarding a hypoxic event, the initiation of therapeutic hypothermia is often recommended. The presence of a thalamic lesion in the presented case indicates potential perinatal hypoxic brain damage in addition to PCH1E. Although cooling would not have changed the outcome for our patient, it could prove beneficial in situations where NE is caused by factors other than HIE and is the cause rather than the consequence of asphyxic brain damage.

The diagnostic journey in NE involves tailored approaches based on clinical presentation. In this case, the association of a history of neonatal death or consanguinity, and the lack of metabolic aberrations, coupled with neuroimaging and neuromuscular findings, prompted pursuit of a neuromuscular etiology. Understanding the nuanced clinical presentation of PCH1E orientates neonatologists in diagnosis and informed decision-making. The clinical observations reported in PCH1E are summarized in Table 1*,* the association of respiratory failure, cerebellar pons hypoplasia and sensory-motor neuropathy being the key features.

**Table 1:** Most common findings in PCH1E children (1, 7–9, 11, 15–17, 19, 20).

Prenatal	Polyhydramnios
Respiratory	Absence of efficient respiratory movements or respiratory distress syndrome^a^
Neurological	Severe hypotonia^a^
	Myoclonic jerks, congenital contractures
	Convulsions
	EEG: global encephalopathy with or without epileptic discharge
	EMG: Axonal sensory motor neuropathy
Brain imaging	Global cerebellar hypoplasia ^a^ + - pons/brainstem hypoplasia
Laboratory	Lactic acidosis, normal CK, liver and respiratory chains enzymes, normal ammonia, normal serum amino acids and serum transferrin

EEG: electroencephalography, EMG: electromyography, CK: creatinine kinase.

^a^ Reported in every PCH1E infant.

Prenatal diagnosis can be challenging; while congenital contractures have occasionally been described at birth, they were not reported during the prenatal care of PCH1E cases (1, 7–9, 11, 15–17, 19, 20). In PCH2, ultrasonography is unreliable for early diagnosis, as characteristic findings of pontocerebellar dysgenesis typically develop after 30 weeks of gestation (21). To our knowledge, anomalies of the posterior fossa, such as a large cisterna magna or a reduction in cerebellar transverse diameter, were not reported during the pregnancy of patients with PCH1E. Although not strongly supported by evidence, this observation suggests a late presentation of abnormalities in PCH1E, akin to PCH2.

Our patient's variant, c.72delT in *SLC25A46*, is novel. No biochemical experiments were conducted to determine the functional impact of the mutation. However, the correlation of our case with the severe clinical phenotype found in the loss-of-function mutations aligns with a class IV variant causing PCH1E. While therapeutic options are currently limited, emerging gene therapy interventions hold promise, as demonstrated in murine models (22).

## Conclusion

This report delineates a novel pathogenic variant c.72delT p. (Phe24LeufsTer20) in the *SLC25A46* gene, expanding the spectrum of genetic alterations linked to PCH1E. Early recognition of the distinct clinical manifestations of PCH1E assists neonatologists in diagnosing and making informed decisions.

## Data Availability

The original contributions presented in the study are included in the article/Supplementary Material, further inquiries can be directed to the corresponding author.

## References

[1] Kurinczuk JJ White-Koning M Badawi N. Epidemiology of neonatal encephalopathy and hypoxic–ischaemic encephalopathy. Early Hum Dev. (2010) 86:329–38. 10.1016/j.earlhumdev.2010.05.010 20554402

[2] Martinello K Hart AR Yap S Mitra S Robertson NJ. Management and investigation of neonatal encephalopathy: 2017 update. Arch Dis Child Fetal Neonatal Ed. (2017) 102:F346–58. 10.1136/archdischild-2015-309639 28389438 PMC5537522

[3] Barth PG. Pontocerebellar hypoplasias. An overview of a group of inherited neurodegenerative disorders with fetal onset. Brain Dev. (1993) 15:411–22. 10.1016/0387-7604(93)90080-r 8147499

[4] McKusick-Nathans Institute of Genetic Medicine, Johns Hopkins University. *Online Mendelian Inheritance in Man*, OMIM®. Available online at: https://omim.org/ (Accessed May 2, 2022)

[5] Sánchez-Albisua I Frölich S Barth PG Steinlin M Krägeloh-Mann I. Natural course of pontocerebellar hypoplasia type 2A. Orphanet J Rare Dis. (2014) 9:70. 10.1186/1750-1172-9-70 24886362 PMC4019562

[6] van Dijk T Baas F Barth PG Poll-The BT. What’s new in pontocerebellar hypoplasia? An update on genes and subtypes. Orphanet J Rare Dis. (2018) 13:92. 10.1186/s13023-018-0826-2 29903031 PMC6003036

[7] Namavar Y Barth PG Poll-The BT Baas F. Classification, diagnosis and potential mechanisms in pontocerebellar hypoplasia. Orphanet J Rare Dis. (2011) 6:50. 10.1186/1750-1172-6-50 21749694 PMC3159098

[8] van Dijk T Rudnik-Schöneborn S Senderek J Hajmousa G Mei H Dusl M Pontocerebellar hypoplasia with spinal muscular atrophy (PCH1): identification of SLC25A46 mutations in the original Dutch PCH1 family. Brain. (2017) 140:e46–e46. 10.1093/brain/awx147 28637197

[9] Wan J Steffen J Yourshaw M Mamsa H Andersen E Rudnik-Schöneborn S Loss of function of SLC25A46 causes lethal congenital pontocerebellar hypoplasia. Brain. (2016) 139:2877–90. 10.1093/brain/aww212 27543974 PMC5840878

[10] Abrams AJ Hufnagel RB Rebelo A Zanna C Patel N Gonzalez MA Mutations in SLC25A46, encoding a UGO1-like protein, cause an optic atrophy spectrum disorder. Nat Genet. (2015) 47:926–32. 10.1038/ng.3354 26168012 PMC4520737

[11] Braunisch MC Gallwitz H Abicht A Diebold I Holinski-Feder E Van Maldergem L Extension of the phenotype of biallelic loss-of-function mutations in SLC25A46 to the severe form of pontocerebellar hypoplasia type I: bRAUNISCH et al. Clin Genet. (2018) 93:255–65. 10.1111/cge.13084 28653766

[12] Fenton TR Kim JH. A systematic review and meta-analysis to revise the fenton growth chart for preterm infants. BMC Pediatr. (2013) 13:59. 10.1186/1471-2431-13-59 23601190 PMC3637477

[13] Madar J Roehr CC Ainsworth S Ersdal H Morley C Rüdiger M European resuscitation council guidelines 2021: newborn resuscitation and support of transition of infants at birth. Resuscitation. (2021) 161:291–326. 10.1016/j.resuscitation.2021.02.014 33773829

[14] Wortmann SB Kluijtmans LAJ Rodenburg RJ Sass JO Nouws J van Kaauwen EP 3-methylglutaconic aciduria—lessons from 50 genes and 977 patients. J Inherit Metab Dis. (2013) 36:913–21. 10.1007/s10545-012-9579-6 23355087

[15] Charlesworth G Balint B Mencacci NE Carr L Wood NW Bhatia KP. SLC25A46 mutations underlie progressive myoclonic ataxia with optic atrophy and neuropathy. Mov Disord. (2016) 31:1249–51. 10.1002/mds.26716 27430653

[16] Hammer MB Ding J Mochel F Eleuch-Fayache G Charles P Coutelier M SLC25A46 mutations associated with autosomal recessive cerebellar ataxia in north African families. Neurodegener Dis. (2017) 17:208–12. 10.1159/000464445 28558379 PMC5540751

[17] Janer A Prudent J Paupe V Fahiminiya S Majewski J Sgarioto N SLC25A46 is required for mitochondrial lipid homeostasis and cristae maintenance and is responsible for leigh syndrome. EMBO Mol Med. (2016) 8:1019–38. 10.15252/emmm.201506159 27390132 PMC5009808

[18] Nguyen M Boesten I Hellebrekers DMEI Mulder-den Hartog NM de Coo IFM Smeets HJM Novel pathogenic SLC25A46 splice-site mutation causes an optic atrophy spectrum disorder. Clin Genet. (2017) 91:121–5. 10.1111/cge.12774 26951855

[19] Abrams AJ Fontanesi F Tan NBL Buglo E Campeanu IJ Rebelo AP Insights into the genotype-phenotype correlation and molecular function of SLC25A46. Hum Mutat. (2018) 39:1995–2007. 10.1002/humu.23639 30178502 PMC6240357

[20] Sulaiman RA Patel N Alsharif H Arold ST Alkuraya FS. A novel mutation in SLC25A46 causes optic atrophy and progressive limb spasticity, with no cerebellar atrophy or axonal neuropathy. Clin Genet. (2017) 92:230–1. 10.1111/cge.12963 28369803

[21] Graham JM Spencer AH Grinberg I Niesen CE Platt LD Maya M Molecular and neuroimaging findings in pontocerebellar hypoplasia type 2 (PCH2): is prenatal diagnosis possible? American J of Med Genetics Pt A. (2010) 152A:2268–76. 10.1002/ajmg.a.33579 PMC2931360 20803644

[22] Yang L Slone J Li Z Lou X Hu Y-C Queme LF Systemic administration of AAV-Slc25a46 mitigates mitochondrial neuropathy in Slc25a46−/− mice. Hum Mol Genet. (2020) 29:649–61. 10.1093/hmg/ddz277 31943007 PMC7068115

